# Association of ten gastrointestinal and other medical conditions with positivity to faecal occult blood testing in routine screening: a large prospective study of women in England

**DOI:** 10.1093/ije/dyy271

**Published:** 2019-01-21

**Authors:** Emily He, Rupert Alison, Roger Blanks, Kirstin Pirie, Gillian Reeves, Robyn L Ward, Robert Steele, Julietta Patnick, Karen Canfell, Valerie Beral, Jane Green

**Affiliations:** 1 Cancer Epidemiology Unit, Nuffield Department of Population Health, University of Oxford, Oxford, UK; 2 Prince of Wales Clinical School, University of New South Wales Sydney, NSW, Australia; 3 Cancer Research Division, Cancer Council NSW, Woolloomooloo, NSW, Australia; 4 Faculty of Medicine and Health, University of Sydney, Sydney, NSW, Australia; 5 Department of Surgery, Ninewells Hospital, Dundee, UK; 6 School of Public Health, University of Sydney, Sydney, NSW, Australia

**Keywords:** Bowel cancer screening, faecal occult blood test, bowel cancer, colonoscopy, upper gastrointestinal bleeding

## Abstract

**Background:**

In 2006, the Bowel Cancer Screening Programme (BCSP) in England began offering biennial faecal occult blood testing (FOBt) at ages 60–69 years. Although FOBt is aimed at detecting colorectal neoplasms, other conditions can affect the result. In a large UK prospective study, we examined associations, both before and after screening, between FOBt positivity and 10 conditions that are often associated with gastrointestinal bleeding.

**Methods:**

By electronically linking BCSP and Million Women Study records, we identified 604 495 women without previous colorectal cancer who participated in their first routine FOBt screening between 2006 and 2012. Regression models, using linked national hospital admission records, yielded adjusted relative risks (RRs) in FOBt-positive versus FOBt-negative women for colorectal cancer, adenoma, diverticular disease, inflammatory bowel disease, haemorrhoids, upper gastrointestinal cancer, oesophagitis, peptic ulcer, anaemia and other haematological disorders.

**Results:**

RRs in FOBt-positive versus FOBt-negative women were 201.3 (95% CI 173.8–233.2) for colorectal cancer and 197.9 (95% CI 180.6–216.8) for adenoma within 12 months after screening and 3.49 (95% CI 2.31–5.26) and 4.88 (95% CI 3.80–6.26), respectively, 12–24 months after screening; *P* < 0.001 for all RRs. In the 12 months after screening, the RR for inflammatory bowel disease was 26.3 (95% CI 19.9–34.7), and ranged between 2 and 5 for the upper gastrointestinal or haematological disorders. The RRs of being diagnosed with any of the eight conditions other than colorectal neoplasms before screening, and in the 12–24 months after screening, were 1.81 (95% CI 1.81–2.01) and 1.92 (95% CI 1.66–2.13), respectively.

**Conclusions:**

Whereas FOBt positivity is associated with a substantially increased risk of colorectal neoplasms after screening, eight other gastrointestinal and haematological conditions are also associated with FOBt positivity, both before and after screening.

**Key Messages**

- In the first 12 months after screening, women who are FOBt-positive have about a 200-fold increase in the risk of colorectal neoplasia. This excess risk remains slightly elevated in the 12–24 months after screening.
- FOBt positivity is associated with an increased risk of being diagnosed with non-neoplastic colorectal conditions that can also cause gastrointestinal bleeding. The greatest relative risk, both before and after FOBt positivity, is for inflammatory bowel disease.
- Bleeding from upper gastrointestinal conditions, especially peptic ulcers, makes a sizeable contribution to FOBt positivity. However, the proportion of FOBt-positive women subsequently diagnosed with upper gastrointestinal cancer is exceedingly low.
- About one in eight women who were FOBt-positive did not undertake follow-up investigations through services provided by the national Bowel Cancer Screening Programme, but many of them had pre-existing conditions associated with gastrointestinal bleeding and appear to have chosen to seek follow-up through other services where they were already under care.

## Background

Colorectal cancer is the third most common cause of cancer deaths in the UK, and accounted for 12% of all new cancers in 2014.^1^ As colorectal cancers are prone to bleeding, the rationale for screening with faecal occult blood test (FOBt) is to detect blood originating from otherwise asymptomatic tumours, thereby enabling diagnosis at an early, potentially curable stage.^2–5^ Although it is acknowledged that FOBt positivity can be affected by a range of conditions other than colorectal cancer,^6^ no previous study has evaluated the associations of FOBt positivity with other gastrointestinal or haematological conditions in a population-based bowel cancer screening programme. Quantifying associations between FOBt positivity and colorectal cancer, as well as other gastrointestinal and haematological conditions, may help guide clinical decision making at both a population and an individual level.

The National Health Service (NHS) Bowel Cancer Screening Programme (BCSP) in England was launched in 2006 to provide biennial colorectal cancer screening using guaiac-based FOBt. In this report, we link FOBt results in the BCSP with information on cause-specific hospital admissions in the large prospective study of women’s health, the Million Women Study. We investigated associations between FOBt positivity and 10 pre-specified conditions related to gastrointestinal bleeding.

## Methods

### The Bowel Cancer Screening Programme in England

The BCSP in England began offering biennial screening with FOBt to men and women aged 60–69 years from 2006 (extended to 74 years from 2010).^7^^,^^8^ Those defined as FOBt-positive by the BCSP were referred to specialist screening nurses at bowel cancer screening centres for further investigations. In most cases, this investigation was a colonoscopy; radiological imaging was performed in the small minority deemed unsuitable for colonoscopy. The BCSP records individual data, including the dates of screening invitations, FOBt results and, for those who were FOBt-positive, findings of further investigations organized by the BCSP.

### The Million Women Study

The Million Women Study is a large nationwide study of women’s health. A total of 1.3 million women, representing one in four of all UK women born in 1935–50, joined the study in 1996–2001. Participants answered a questionnaire on sociodemographic, health and lifestyle characteristics, and consented to follow-up through medical records. Details of the study design have been published elsewhere.^9^^,^^10^ Questionnaires and information on data access for the study can be viewed at www.millionwomenstudy.org.

Using their unique NHS number and other identifying details, study participants are followed by linkage to electronic, routinely collected NHS records for deaths, cancer registrations and hospital admissions. Data for England are provided through NHS Digital. The Hospital Episode Statistics (HES) inpatient dataset in England contains details of all NHS inpatient (overnight and day case) admissions, including private patients treated in NHS hospitals. Diagnoses are recorded using the Tenth Revision of the International Classification of Diseases (ICD-10).^11^ Colonoscopies are generally performed as ‘day cases’ in NHS hospitals, and their findings are recorded in the HES database. Cancer registration, death and emigration records are available from recruitment to 31 December 2014 and HES records from 1 April 1997 to 31 December 2014.

With approval from the Cambridge South Research Ethics Committee and by the NHS Bowel Cancer Screening Programme Research Committee, the BCSP database was linked to the Million Women Study database in 2013 (done by NHS Connecting for Health, now NHS Digital).

### Outcomes

Linked HES records were searched for hospital admission with diagnoses of the following 10 pre-specified gastrointestinal and haematological conditions: colorectal cancer, colorectal adenoma, inflammatory bowel disease, diverticular disease, haemorrhoids, peptic ulcer disease, oesophagitis, upper gastrointestinal cancer, anaemia and other bleeding disorders. Table 1 gives the ICD-10 codes used to define each of the 10 conditions. More than one diagnosis may have been recorded in a single hospital admission. We did validation analyses for colorectal cancer using cases ascertained from the cancer registry data.^8^

**Table 1.** Classification of HES diagnoses using ICD-10

Diagnosis in analysis	**ICD-10** ^a^	Description
Colorectal cancer	C18	Malignant neoplasm of colon
	C19	Malignant neoplasm of rectosigmoid junction
	C20	Malignant neoplasm of rectum
Colorectal adenoma	D12	Benign neoplasm of colon, rectum, anus or anal canal
Inflammatory bowel disease	K50	Crohn’s disease [regional enteritis]
	K51	Ulcerative colitis
Diverticular disease	K57	Diverticular disease of intestine
Haemorrhoids	I84	Haemorrhoids
Peptic ulcer disease	K25	Gastric ulcer
	K26	Duodenal ulcer
	K27	Peptic ulcer, site unspecified
	K28	Gastrojejunal ulcer
	K29	Gastritis and duodenitis
Oesophagitis	K20	Oesophagitis
	K21	Gastro-oesophageal reflux disease
Upper gastrointestinal cancer	C15	Malignant neoplasm of oesophagus
	C16	Malignant neoplasm of stomach
	C17	Malignant neoplasm of small intestine
Anaemias	D50	Iron deficiency anaemia
	D51	Vitamin B12 deficiency anaemia
	D52	Folate deficiency anaemia
	D53	Other nutritional anaemias
Haematological conditions	D65-D69	Coagulation defects, purpura and other haemorrhagic conditions
	D70-D77	Other diseases of blood and blood-forming organs
	C81-C96	Malignant neoplasm of lymphoid, haematopoietic and related tissue

a First three characters of ICD-10 codes were used to define diagnoses used in the analysis.

Conditions were defined as being diagnosed ‘before screening’ if the first hospital record for the condition was before the first BCSP screening invitation. Conditions were defined as being diagnosed ‘after screening’ if the first hospital record of the condition was after the first screening invitation. First diagnoses after screening were further divided into those in the first 12 months after screening invitation (i.e. including results of diagnostic investigations following a positive screening test) and those first recorded in the 12–24 months after screening. Diagnoses after 24 months were not included, as the BCSP would have offered many women routine screening again 24 months after the first invitation. For FOBt-positive patients who underwent subsequent colonoscopy, the information was obtained from hospital diagnoses in the HES database, as colonoscopies are generally performed as inpatient ‘day cases’ in NHS hospitals.

### Statistical analysis

Record linkage identified 899 166 Million Women Study participants who received their first invitation for routine bowel cancer screening from the BCSP (prevalent screening round). For these analyses, we excluded women who did not accept the invitation for screening (*n* = 278 705), had any solid or haematological malignancy registered before recruitment into Million Women Study (*n* = 15 144), were lost to follow-up before first BCSP invitation (*n* = 22) or had a hospital record of colorectal cancer before FOBt screening (*n* = 800). The remaining 604 495 participants, first screened for bowel cancer between 2006 and 2012, were included in the analyses.

For conditions first diagnosed before screening, logistic regression models estimated odds ratios, henceforth referred to as relative risks (RRs), for various diagnoses in FOBt-positive versus FOBt-negative women. For conditions first diagnosed after screening, Cox regression models, with attained age as the underlying time variable, estimated hazard ratios, henceforth also referred to as relative risks (RRs), for first hospital diagnoses in FOBt-positive versus FOBt-negative women in the first 12 months after screening, and 12–24 months after screening. For every RR estimate, 95% confidence intervals (CI) were calculated.

All analyses were adjusted for area deprivation (based on the Townsend index^12^), smoking (never, past, current), alcohol consumption in drinkers (<2, 3–14, ≥15 drinks per week) and body mass index (<25, 25–29, 30+ kg/m^2^), as these have been associated increased risk of colorectal cancer. Information relating to these variables was obtained at recruitment; women with missing data on adjustment variables (<2% for each) were included in the analyses as a separate category. Information on family history of colorectal cancer and use of antiplatelet or anticoagulant medications were not collected at recruitment. Analyses of diagnoses other than colorectal neoplasms (cancer or adenoma) excluded women with colorectal cancer diagnosed in the 24 months after screening. All analyses were performed using Stata version 14.0.

## Results

Of the 604 495 women without previous colorectal cancer who had a routine FOBt screen, 8852 (1.5%) were FOBt-positive and referred for further investigations in the Bowel Cancer Screening Programme (BCSP). All FOBt screening was performed between 13 July 2006 and 13 March 2012.

The baseline characteristics and the frequency of conditions recorded in hospital admission data before the FOBt screen are shown in Table 2. Results are given for nine of the 10 gastrointestinal and haematological conditions being investigated here, as the 10th condition is colorectal cancer, and women with previous colorectal cancer were excluded from these analyses.

**Table 2.** Baseline characteristics and pre-screening HES diagnoses for hospital admissions in FOBt-positive women (*n *= 8852) versus FOBt-negative women (*n* = 595 643)

Participant characteristics	FOBt-positive (*n* = 8 852), % (*n*)	FOBt-negative (*n* = 595 643), % (*n*)
Mean age at first invitation to bowel screening, years (SD)	65.9 (3.7)	65.3 (3.6)
Socioeconomic group (% in upper third)	31.2% (2757)	36.0% (14 377)
Current smoker	18.0% (1596)	15.8% (93 980)
Body mass index ≥30 kg m^2^	23.7% (2100)	15.1% (89 618)
Alcohol intake ≥30 g per week	41.3% (3655)	43.5% (258 826)
Hospital admission before routine FOB testing for nine pre-specified conditions^a^		
Colorectal neoplasms		
Colorectal adenomas	1.97% (174)	0.93% (5562)
Colorectal non-neoplastic		
Inflammatory bowel disease	1.79% (146)	0.27% (1614)
Diverticular disease	6.27% (511)	3.19% (18 975)
Haemorrhoids	5.12% (417)	2.54% (15 131)
Upper gastrointestinal		
Oesophagitis	6.99% (570)	4.37% (26 009)
Peptic ulcer	9.29% (757)	5.24% (31 177)
Upper gastrointestinal cancer	0.15% (12)	0.06% (332)
Haematological		
Anaemia	3.86% (315)	0.82% (4867)
Potential bleeding tendency	6.43% (524)	2.16% (12 880)
Any of the above conditions, except colorectal neoplasms	25.2% (2007)	15.6% (82 677)

a Women with the 10th condition being investigated, colorectal cancer, were excluded from these analyses if the cancer had been diagnosed before routine screening.

Before screening, FOBt-positive women were more likely than FOBt-negative women to have had previous diagnoses of colorectal adenoma or non-neoplastic colorectal conditions (inflammatory bowel disease, diverticular disease and haemorrhoids), as well as upper gastrointestinal conditions (oesophagitis, peptic ulcer disease and upper gastrointestinal cancer). FOBt-positive women were also more likely than FOBt-negative women to have had a previous admission for anaemia or other haematological disorders. The proportions of women admitted with at least one of the eight conditions (other than colorectal adenoma) before FOB testing were 25% of FOBt-positive and 16% of FOBt-negative participants. The pre-screening diagnoses most strongly associated with FOBt positivity were inflammatory bowel disease (RR = 6.06, 5.11 – 7.18; *P* <0.0001) and anaemia (RR = 4.20, 3.74 – 4.71; *P* <0.0001).

Of the FOBt-positive women, 7687 (87%) attended the BCSP for further investigations, which for almost all women was a colonoscopy. Among the 1174 (13%) FOBt-positive women who did not attend the BCSP for further investigation, hospital admission records indicate that they were significantly more likely than the attenders to have had previous hospital admissions for each of the nine pre-selected gastrointestinal and haematological conditions, particularly inflammatory bowel disease and anaemia (Table 3).

**Table 3.** Pre-screening diagnoses in FOBt-positive women^a^ by attendance at Bowel Cancer Screening Programme for further diagnostic tests

	FOBt-positive who attended BCSP for further diagnostic tests (*n* = 7678)		FOBt-positive who did not attend BCSP for further diagnostic tests (*n* = 1174)	
	% (*n*)	Adjusted RR^b^ (95% CI)	% (*n*)	Adjusted RR^b^ (95% CI)
Colorectal neoplasms				
Colorectal adenoma	1.52% (117)	1.49 (1.24 – 1.79)	4.86% (57)	4.62 (3.53 – 6.04)
Colorectal non-neoplastic				
Inflammatory bowel disease	0.82% (63)	2.93 (2.28 – 3.78)	7.33% (86)	27.2 (21.66 – 34.08)
Diverticular disease	5.25% (403)	1.48 (1.34 – 1.64)	10.2% (120)	2.92 (2.41 – 3.53)
Haemorrhoids	4.39% (337)	1.68 (1.51 – 1.88)	7.92% (93)	3.10 (2.5 – 3.83)
Upper gastrointestinal				
Oesophagitis	6.90% (530)	1.48 (1.35 – 1.62)	7.16% (84)	1.48 (1.19 – 1.85)
Peptic ulcer disease	8.44% (648)	1.53 (1.41 – 1.66)	12.0% (141)	2.15 (1.8 – 2.56)
Upper gastrointestinal cancer	0.13% (10)	2.17 (1.15 – 4.07)	0.17% (2)	2.70 (0.67 – 10.88)
Haematological				
Anaemia	2.90% (223)	3.34 (2.92 – 3.83)	8.43% (99)	9.97 (8.09 – 12.3)
Potential bleeding tendency	4.98% (382)	2.19 (1.97 – 2.43)	13.3% (156)	6.20 (5.22 – 7.35)
Any of the above, except colorectal neoplasms	22.2% (1681)	1.62 (1.53 – 1.71)	38.0% (424)	3.39 (3.00 – 3.83)

a Women with the 10th condition being investigated, colorectal cancer, were excluded from these analyses if the cancer had been diagnosed prior to routine screening.

b Relative risk in FOBt-positive versus FOBt-negative women, adjusted for age, socioeconomic group, smoking status, alcohol intake and body mass index.

In all, 700 (7.9%) FOBt-positive women had a hospital diagnosis for colorectal cancer and 1704 (19%) for colorectal adenoma in the first 12 months after screening. Among the FOBt-negative women, the corresponding numbers were 247 and 649, yielding relative risks of 201.3 (173.8 – 233.2) and 197.9 (180.6 – 216.8) for colorectal cancer and for adenoma in FOBt-positive compared with FOBt-negative women. Results for colorectal cancer were similar, based on cancers ascertained from the cancer registry data, at 212.0 (184.0 – 246.5).

**Figure 1:**
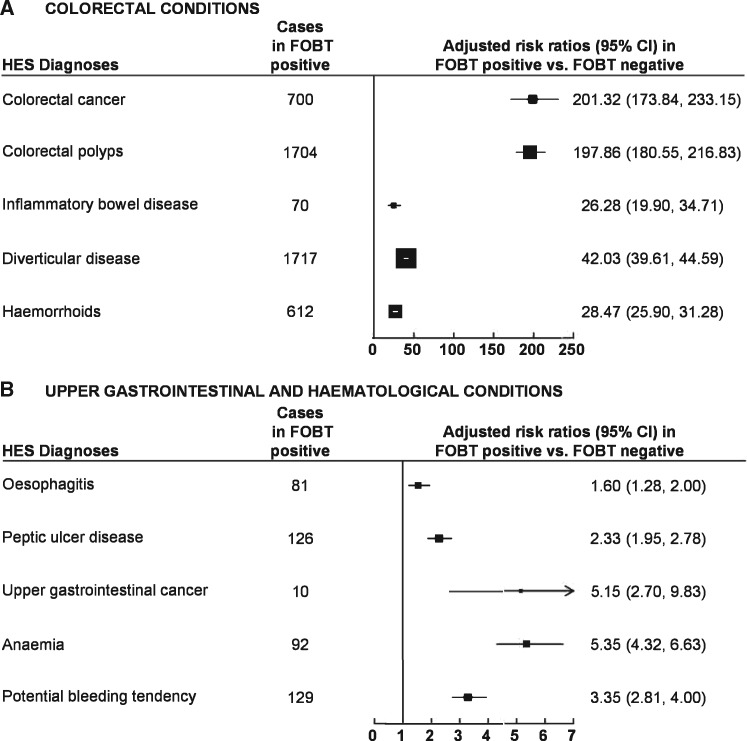
Risk ratios for various conditions first diagnosed in the 12 months after positive FOBt. [A] shows the risk ratios for colorectal conditions. [B] shows the risk ratios for upper gastrointestinal and haematological conditions. Note different scales are used in (A) and (B) in the figure.

In the first 12 months after screening, FOBt positivity was also associated with large increases in non-neoplastic colorectal conditions, with 1717 FOBt-positive women diagnosed with diverticular disease (RR = 42.0, 39.6 – 44.6), 612 with haemorrhoids (RR = 28.5, 25.9 – 31.3) and 70 with inflammatory bowel disease (RR = 26.3, 19.9 – 34.7); *P* <0.0001 for all the RRs quoted (Figure 1A). Virtually all the colorectal conditions (both neoplastic and non-neoplastic) were recorded in the 6 months after the FOBt screening invitation and were probably diagnosed as a result of the BCSP referral.

After excluding those diagnosed with colorectal cancer after screening, FOBt positivity was associated with a 5-fold risk of upper gastrointestinal cancer and 2-fold risk of peptic ulcer or oesophagitis in the first 12 months after screening (Figure 1B). It was also associated with a 3–5-fold relative risk of anaemia or other bleeding tendency being diagnosed in the first 12 months after screening.

Women who attended the BCSP for diagnostic colonoscopies had higher relative risks for colorectal cancer and adenoma, although those who did not attend also had considerably raised risks for both (Table 4). The RRs for colorectal cancer in attenders and non-attenders were 220.5 (190.2 – 255.6) and 74.7 (52.8 – 105.6), respectively, and the corresponding RRs for colorectal adenoma were 228.3 (208.3 – 250.2) and 21.1 (14.6 – 30.4), respectively; *P* <0.0001 for all the RRs quoted. In validation analysis, the RRs for colorectal cancer in attenders and non-attenders, using diagnoses ascertained from the cancer registry data, were similar to those based on HES data, at 232.0 (201.1 – 269.9) and 78.4 (55.8 – 110.1), respectively. The mean time between FOBt screening invitation and HES record of colorectal cancer diagnosis was also similar in the 662 attenders and 38 non-attenders, at 3.0 [standard deviation (SD) 1.6] and 4.9 (SD 2.9) months, respectively. For upper gastrointestinal cancer and anaemia, RRs in the first 12 months after screening were significantly greater in non-attenders than in attenders for BCSP investigations.

**Table 4.** Conditions diagnosed in hospital admission records for the first time in the initial 12 months after routine FOB testing in FOBt-positive women, by attendance at Bowel Cancer Screening Programme for further diagnostic tests

Diagnosis in HES	FOBt-positive who attended BCSP for further diagnostic tests (*n* = 7678)		FOBt-positive who did not attend BCSP for further diagnostic tests (*n* = 1174)	
	% (*n*)	Adjusted RR^a^ (95% CI)	% (*n*)	Adjusted RR^a^ (95% CI)
Colorectal neoplasms				
Colorectal cancer	8.62% (662)	220.5 (190.2 – 255.6)	3.24% (38)	74.7 (52.8 – 105.6)
Colorectal adenoma	21.8% (1674)	228.3 (208.3 – 250.2)	2.56% (30)	21.1 (14.6 – 30.4)
Colorectal non-neoplastic^b^				
Inflammatory bowel disease	0.84% (59)	25.7 (19.1 – 34.6)	0.98% (11)	28.8 (15.6 – 53.1)
Diverticular disease	23.9% (1671)	49.1 (46.2 – 52.1)	3.72% (42)	5.99 (4.42 – 8.13)
Haemorrhoids	8.31% (582)	31.7 (28.8 – 34.8)	2.66% (30)	9.45 (6.58 – 13.56)
Upper gastrointestinal^b^				
Oesophagitis	0.93% (65)	1.49 (1.17 – 1.91)	1.42% (16)	2.25 (1.38 – 3.68)
Peptic ulcer	1.49% (104)	2.24 (1.84 – 2.72)	1.95% (22)	2.86 (1.88 – 4.35)
Upper gastrointestinal cancer	0.09% (6)	3.59 (1.58 – 8.16)	0.35% (4)	14.1 (5.21 – 38.4)
Haematological^b^				
Anaemia	0.87% (61)	4.13 (3.19 – 5.36)	2.75% (31)	12.4 (8.7 – 17.8)
Potential bleeding tendency	1.40% (98)	2.97 (2.43 – 3.64)	2.75% (31)	5.59 (3.92 – 7.97)
Any of the above, except colorectal neoplasms	37.4% (2577)	24.2 (23.1 – 25.2)	12.6% (135)	5.48 (4.62 – 6.49)

a Relative risk in FOBt-positive versus FOBT-negative women, adjusted for age, socioeconomic group, smoking status, alcohol intake and body mass index.

b Women who were diagnosed with colorectal cancer within 24 months after routine FOBt were excluded from analyses relating to diagnoses other than cancer.

**Figure 2.**
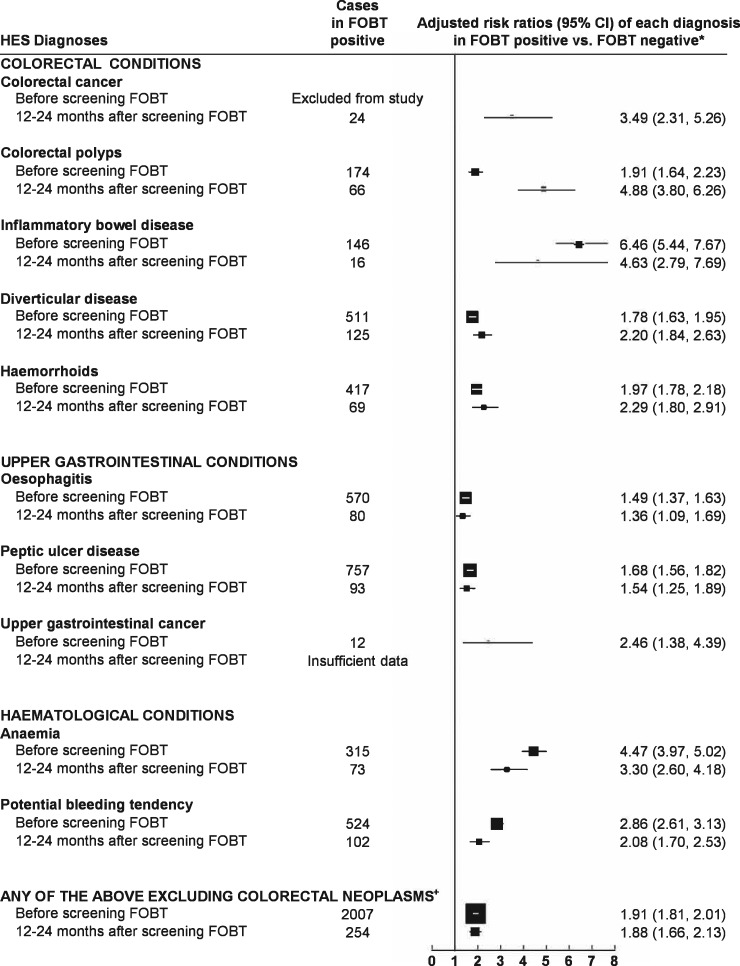
Risk ratios for colorectal, upper gastrointestinal and haematological conditions first diagnosed before FOBt and those first diagnosed 12–24 months after positive FOBt. *First hospital admission 12–24 months after FOBt, adjusted for socioeconomic group, body mass index, alcohol consumption and smoking status. ‘Colorectal neoplasms’ encompasses colorectal cancer and colorectal polyps.

In the 12–24 months after screening, the relative risks for colorectal cancer (RR = 3.49, 2.31 – 5.26; *P* <0.0001) and for colorectal adenoma (RR = 4.88, 3.80 – 6.26; *P* <0.0001) remain elevated in in FOBt-positive versus FOBT-negative women, although much lower than in the preceding 12 months (for colorectal cancer, the numbers were 24 and 475, respectively; and for colorectal adenoma, the numbers were 66 and 1075, respectively). There was also an increased risk of being diagnosed with other colorectal, upper gastrointestinal and haematological conditions (Figure 2). The RRs of being diagnosed with any of the eight conditions other than colorectal neoplasms were about doubled, both before screening and in the 12–24 months after screening, at 1.81 (1.72 – 1.90) and 1.92 (1.70 – 2.17), respectively.

## Discussion

Many countries, such as the UK and Australia, have implemented population-based screening programmes using FOBt.^13^ However, conditions other than colorectal neoplasms can affect the test result, and the specificity of FOBt has been found to be variable.^14^ This is the first study to examine systematically, in a large cohort of 604 495 women who took part in routine bowel cancer screening in England, associations between FOBt positivity and clinical conditions related to gastrointestinal bleeding, both before and after screening.

In this study, 1.5% of those screened were FOBt-positive, and 7.9% of those who were FOBt-positive were diagnosed with colorectal cancer in the first 12 months after screening. These findings are consistent with previous estimates reported by the UK Bowel Cancer Screening Programme.^8^^,^^15^^,^^16^ Compared with FOBt-negative women, FOBt-positive women had a 200-fold greater risk of being diagnosed with colorectal neoplasms in the first 12 months after screening, falling to a 3–5-fold relative risk in the 12–24 months after screening. The findings here relate to the prevalent round of colorectal cancer screening and the positive predictive value of screening has been reported to be reduced after the prevalent screen.^17–20^ The excess risk of colorectal neoplasms and of other conditions in the first 12 months after FOBt positivity reflects, in large part, that FOBt-positive individuals are offered further investigations by the BCSP whereas FOBt-negative individuals are not.

Almost 30% of the FOBt-positive women in this study had a hospital diagnosis of a non-neoplastic colorectal condition (mainly diverticular disease or haemorrhoids) in the 12 months after FOBt. Virtually all the diagnoses were made at around the time of colonoscopy, which is generally recorded as day case hospital admission. Our findings are in line with results reported by other organised screening programmes, that 24–38% of FOBt-positive individuals have non-neoplastic colorectal conditions identified during diagnostic colonoscopies.^15^^,^^21^^,^^22^ Although diverticular disease has been reported to account for some 40% of clinical lower gastrointestinal bleeding episodes,^23^ it is generally considered an incidental finding in colonoscopy for investigation of positive FOBt.^24^

About 15% of FOBt-positive women had a hospital admission with benign causes of upper gastrointestinal bleeding before screening and in 1% this was first diagnosed in the 12 months after screening, consistent with previous findings.^25–28^ The majority of these conditions were non-malignant, mostly peptic ulcer disease. In contrast, the proportion of FOBt-positive women diagnosed with upper gastrointestinal cancer (although statistically significantly higher than in FOBt-negative women) is exceedingly low, present in only about 0.1% of FOBt-positive women before testing and a further 0.1% in the next 12 months. Other limited evidence indicate that considerably fewer than 1% of FOBt-positive colonoscopy-negative patients have upper gastrointestinal cancer.^28–30^

The relative risks of being diagnosed with anaemia (>95% were iron deficiency anaemia) in FOBt-positive versus FOBt-negative women, before and after screening, range from 3 to 5. The anaemia itself may not necessarily be caused by gastrointestinal bleeding but could be a manifestation of some other underlying haematological condition. Approximately 6% of the FOBt-positive women had a previous hospital diagnosis of other specified haematological conditions which could predispose to gastrointestinal bleeding, and about 1.2% had a first diagnosis of one of these conditions in the 12 months after FOB testing. The associated relative risks are approximately 3–4, suggesting that the presence of these conditions influences FOBt positivity. A small part of the excess risks for non-colorectal conditions diagnosed after screening could be due to increased contact with health professionals.

About one in eight who were FOBt-positive did not attend further investigations organised by the BCSP. Non-attenders were more likely than the attenders to have the pre-existing conditions associated here with gastrointestinal bleeding. Some have suggested that the most common reasons for non-attendance at BCSP after FOBt positivity include unwillingness to undergo a colonoscopy or inopportune timing,^31^ but our results suggest that many had pre-existing conditions associated with gastrointestinal bleeding and appeared to have diagnostic investigations done elsewhere, possibly because they were already under the care of other clinicians within the NHS.

The findings here are for a population-based NHS screening programme using guaiac-based FOBt, and may not be directly translatable to other screening programmes that use immunochemical-based FOBt (faecal immunochemical testing, FIT). The characteristics of screen-detected and interval cancers are being reported elsewhere.^32^

Some of the important strengths of this study include having a virtually complete dataset on cause-specific NHS hospital admissions, both before and after FOBt screening. By linking BCSP data to information collected in the Million Women Study, it was possible to obtain information on clinical diagnoses recorded before screening as well as after screening, not only for the 87% of the FOBt-positive women who attended the BCSP for further investigations, but also for the 13% who did not. The main limitation of this study is that diagnoses are restricted to those associated with hospital admissions (overnight or day case stays) and that it involved only women, who have lower rates of FOBt positivity and lower risks of colorectal cancer and adenoma compared with men. A systematic review has found the accuracy of primary diagnosis codes in these hospital data to be 96% after 2002.^33^ Some participants may have had outpatient or primary care diagnoses for conditions such as diverticular disease or haemorrhoids, which although amenable to colonoscopic detection, could also have been diagnosed through radiological or clinical examinations.

## Conclusion

Our study confirms that FOBt positivity in routine bowel cancer screening is associated with a substantially increased risk of colorectal neoplasms diagnosed in the first 12 months after screening, and a small excess risk in the 12–24 months after screening. It also showed that FOBt positivity is associated with eight other gastrointestinal or haematological conditions related to gastrointestinal bleeding. These results emphasise the importance of reviewing relevant medical history and discussion about other conditions associated with gastrointestinal bleeding might be considered when counselling patients with positive FOBt results.

## Funding

This was work was supported by the UK Medical Research Council and Cancer Research UK, as well as the White-Walker Cancer Research Scholarship and an Australian National Health and Medical Research Council Postgraduate Scholarship (GNT1093776) for E.H. The study sponsors had no part in designing the study question nor in the analysis or interpretation of results. They also did not take part in the writing of the article nor in the decision to submit for publication.

## Author Contributions

E.H. performed the literature search, participated in the study design and electronic data linkage and was responsible for statistical analysis, data interpretation and drafting of the manuscript. R.A., R.B., K.P. and G.R. participated in the study design, electronic data linkage and statistical analysis. R.W., R.S., J.P. and K.C. provided expert content advice and assisted in interpretation of study findings. J.G. and V.B. oversaw the study, including the study design, statistical analysis and drafting of the manuscript. All authors reviewed the study’s findings and read and approved the final manuscript.

**Conflict of interest:** None declared.
